# Student-led clinics and ePROs to accelerate diagnosis and treatment of patients with axial spondyloarthritis: results from a prospective pilot study

**DOI:** 10.1007/s00296-023-05392-5

**Published:** 2023-07-24

**Authors:** Sophie von Rohr, Johannes Knitza, Manuel Grahammer, Marc Schmalzing, Sebastian Kuhn, Georg Schett, Andreas Ramming, Hannah Labinsky

**Affiliations:** 1 Department of Internal Medicine 3, Rheumatology and Immunology, Friedrich-Alexander University Erlangen-Nürnberg and Universitätsklinikum Erlangen, Erlangen, Germany; 2 Deutsches Zentrum für Immuntherapie, Friedrich-Alexander University Erlangen-Nürnberg and Universitätsklinikum Erlangen, Erlangen, Germany; 3 Université Grenoble Alpes, AGEIS, Grenoble, France; 4 Abaton GmbH, Berlin, Germany; 5 Department of Internal Medicine 2, Rheumatology/Clinical Immunology, University Hospital Würzburg, Oberdürrbacher Straße 6, Würzburg, Germany; 6 Institute for Digital Medicine, University Hospital of Giessen and Marburg, Marburg, Germany; 7 Faculty of Health Sciences Brandenburg, Center for Health Services Research, Brandenburg Medical School Theodor Fontane, Rüdersdorf bei Berlin, Germany

**Keywords:** Axial spondyloarthritis, axSpA, Diagnostic delay, Student, Electronic patient-reported outcome, ePRO

## Abstract

We aimed to investigate (1) student-led clinics and (2) electronic patient-reported outcomes (ePROs) to accelerate diagnosis and treatment of patients with axial spondyloarthritis (axSpA). Patients with suspected axSpA completed an initial student-led clinic visit (T-1) prior to their planned actual rheumatologist visit (T0). Acceleration of patient appointment and NSAID therapy start, availability of diagnostic findings, and treatment response at T0 were evaluated. Beginning at T-1, patients completed electronic BASDAI questionnaires every 2 weeks. Concordance of paper-based and electronic BASDAI was evaluated. Patient acceptance of ePRO reporting and student-led clinics was measured using the net promoter score (NPS). 17/36 (47.2%) included patients were diagnosed with axSpA. Student-led clinics (T-1) significantly accelerated patient appointments by more than 2 months (T0, T-1, *p* < 0.0001) and axSpA guideline-conform NSAID treatment (*p* < 0.0001). At T0, diagnostic workup was completed for all patients and 7/17 (41.2%) axSpA patients presented with a clinically important improvement or were in remission. 34/36 (94.4%) patients completed at least 80% of the ePROs between T-1 and T0. Electronic and paper-administered BASDAI correlated well (r = 0.8 *p* < 0.0001). Student-led clinics and ePROs were well accepted by patients with NPS scores of + 62.0% (mean ± SD 9.2/10.0 ± 0.9) and + 30.5% (mean ± SD 8.0/10.0 ± 1.7), respectively. In conclusion, student-led clinics and ePRO monitoring were well accepted, accelerated diagnostic workup and treatment in patients with axSpA.

**Supplementary Information:**

The online version contains supplementary material available at 10.1007/s00296-023-05392-5.

## Introduction

Axial Spondyloarthritis (axSpA) is one of the more common inflammatory rheumatic diseases with an estimated prevalence of 0.3–1.4% [1, 2]. Despite the availability of improved diagnostic aids and various health service strategies [3], the diagnostic delay of axSpA patients does not decline with around 7 years in Europe [4, 5]. This diagnostic delay often results in irreversible damage, worse treatment, and overall worse prognosis [6] and quality [7] of life. The increasing shortage of rheumatologists is likely to create an even greater diagnostic delay [8].

As a rapid expansion of the rheumatology workforce seems unlikely, innovative new health care strategies are needed. Delegation of tasks and implementation of telehealth are being increasingly discussed and adopted as two main methods to counteract the workforce shortage [9]. Delegation of tasks to specialized rheumatology assistants is increasing, and studies report high patient acceptance [10] and non-inferiority compared to standard care [11]. In other disciplines, e.g., diabetology and hypertensiology, student-led clinics also relieved the health care system and improved patient health outcomes [12]. The added value of student-led clinics in rheumatology is currently unclear.

Electronic patient-reported outcomes (ePROs) enable a flexible and yet standardized disease monitoring. A recent study reported a higher adherence in axSpA patients with high disease activity and a decline after time, suggesting that especially newly diagnosed patients would be motivated to adhere. With regard to the recommended initial therapy with NSAIDS [13], ePRO monitoring could be used to actually guide therapeutic decisions, i.e., switch to another NSAID or escalation of treatment.

Our study aimed to investigate two innovative health services, including (1) the implementation of student-led clinics and (2) electronic patient-reported outcomes to accelerate diagnosis and treatment of patients with axial spondyloarthritis (axSpA).

## Methods

### Study design

This prospective study was approved by the institutional review board (IRB) of the Medical Faculty of the University of **Erlangen-Nürnberg, Germany ** (21–357-B) and conducted in compliance with the Declaration of Helsinki. During the recruitment period (October 2021–July 2022), patients referred by their (primary care) physician for rheumatologic evaluation due to the leading symptom chronic low back pain for at least 3 months and suspected axSpA were recruited. Further inclusion criteria were a minimum age of 18 years, sufficient language skills, and regular usage of a smartphone. Exclusion criteria were a known diagnosis, a previous rheumatologist appointment and unwillingness or inability to comply with the protocol. All study patients provided written informed consent prior to study participation.

Patients completed the student-led appointment (T-1) with one medical student trained for this purpose, prior to the routine rheumatology appointment (T0). Availability of medical findings was assessed at T-1 and at the actual visit with the rheumatologist (T0). Additionally, patients installed a medical app to answer disease activity questionnaires and answered a questionnaire regarding diagnostic delay. Patient acceptance was measured using the net promoter score [14] (NPS), which is based on an 11-point numeric rating scale (0–10). Answers between 0 and 6 are categorized as detractors, 7 and 8 as passives, and 9 and 10 as promoters. The NPS is equal to the percentage of promoters subtracting the percentage of detractors.

### Student-led clinics (T-1)

A medical student independently studied axSpA disease and shadowed rheumatology residents in dedicated axSpA clinics to learn how to carry out a standardized evaluation and axSpA diagnosis. The student then documented the medical history in a standardized fashion, performed a standardized physical examination at T-1, and was given access to laboratory and imaging results once available. After each diagnostic step, the student had to state if axSpA was present or not (yes/no). Results were compared to the final diagnosis reported on the discharge summary report. A rheumatology resident reviewed the results and discussed next steps (start of therapy, further diagnostics) with the patient and student.

### Electronic Patient-Reported Outcomes (ePRO)

At T-1, a medical app (ABATON) was installed on the patient’s smartphone and patients were asked to complete the Bath Ankylosing Spondylitis Disease Activity Index (BASDAI) [15] electronically and on paper at T-1 and hence electronically every 2 weeks. If the patient forgot to answer the questionnaire, he was reminded to answer on 3 consecutive days. Concordance of electronic and paper BASDAI results and adherence were analyzed. At T0, the physician was given access to BASDAI results, enabling a standardized evaluation of NSAID treatment response started at T-1.

### Statistical analysis

No formal sample size calculation was performed due to the exploratory character of the trial. Following recommendations for pilot studies [16], the number of patients was set at 40. Statistical analysis was performed using Microsoft Excel 2019 and GraphPad Prism 8. P values less than 0.05 were considered significant. Patient-to-patient comparisons were summarized by median and interquartile range *(IQR, interquartile range 25*^*th*^* and 75*^*th*^* percentiles) for interval data and as absolute (n) and relative frequency (percent) for nominal data*. Statistical differences were assessed by Mann–Whitney U test, Kruskal–Wallis test with Dunn’s test for multiple comparisons, Spearman correlation analysis (r_s_), and Fisher's exact test for categorial variables. Results were reported following the STAndards for the Reporting of Diagnostic accuracy studies guideline [17]. Diagnostic accuracy of the medical student was evaluated referring to sensitivity, specificity, and overall accuracy.

## Results

17/36 (47.2%) of patients were diagnosed with axSpA. Three patients were lost to follow up due to missed appointments and one patient refused to participate. Baseline patient characteristics are shown in Table 1. Median age was 37.2 years; 21/36 (58.3%) were female.

**Table 1:** Patient characteristics

Patient characteristics	All patients (*n* = 36)	axSpA (*n* = 17)	no axSpA (*n* = 19)	*p* value
Age, Mdn (IQR)	37.2 (20.6)	34.4 (15.2)	39.9 (18.0)	0.24
BMI, Mdn (IQR)	24.6 (5.1)	25.8 (2.3)	24.4 (8.2)	0.59
Active smoker status, N (%)	5 (13.9)	4 (21.1)	1 (5.9)	0.34
Female gender, N (%)​	21 (58.3)	7 (41.2)	14 (73.7)	0.09
Chronic lower back pain, N (%)	36 (100)	17 (100)	19 (100)	1.00
Peripheral enthesopathy, N (%)	10 (27.8)	6 (35.3)	4 (21.1)	0.46
Peripheral arthralgia	15 (41.7)	6 (35.3)	9 (47.4)	0.52
Elevated baseline CRP, N (%)	7 (38.9)	4 (23.5)	3 (15.8)	0.68
HLA-B27 positive, N (%)	18 (50)	10 (58.8)	8 (42.1)	0.51
History of uveitis, N (%)	0 (0)	0 (0)	0 (0)	1.00
History of IBD, N (%)	0 (0)	0 (0)	0 (0)	1.00
History of psoriasis, N (%)	1 (2.8)	1 (5.9)	0 (0)	0.48
NSAR response, N (%)	27 (75)	15 (88.2)	12 (63.2)	0.13
Family history of axSpA	7 (38.9)	1 (5.9)	6 (31.6)	0.09
Family history of IBD	3 (8.3)	0 (0)	3 (15.8)	0.23
Family history of psoriasis	4 (11.1)	1 (5.9)	3 (15.8)	0.61
Baseline PtGA, Mdn (IQR)	5 (3)	4 (3)	5 (3)	0.36
Baseline Morning stiffness at T-1, Mdn (IQR)	30 (51.3)	30 (50)	30 (37.5)	0.86
Baseline BASDAI, Mdn (IQR)	4.2 (2.7)	4 (2.3)	4.3 (2.7)	0.47
BASMI > 0, N (%)	13 (36.1)	6 (35.3)	7 (36.8)	1.00

Statistical significances between the axSpA and non-axSpA patients were determined by Mann–Whitney U test for nominal variables and Fisher’s exact test for categorial variables

*Mdn* Median, *IQR* interquartile range, *BMI* body mass index, *IBD* inflammatory bowel disease, *VAS* visual analogue scale, *BASDAI* Bath Ankylosing Spondylitis Disease Activity Index, *BASFI* Bath Ankylosing Spondylitis Functional Index

**Fig. 1:**
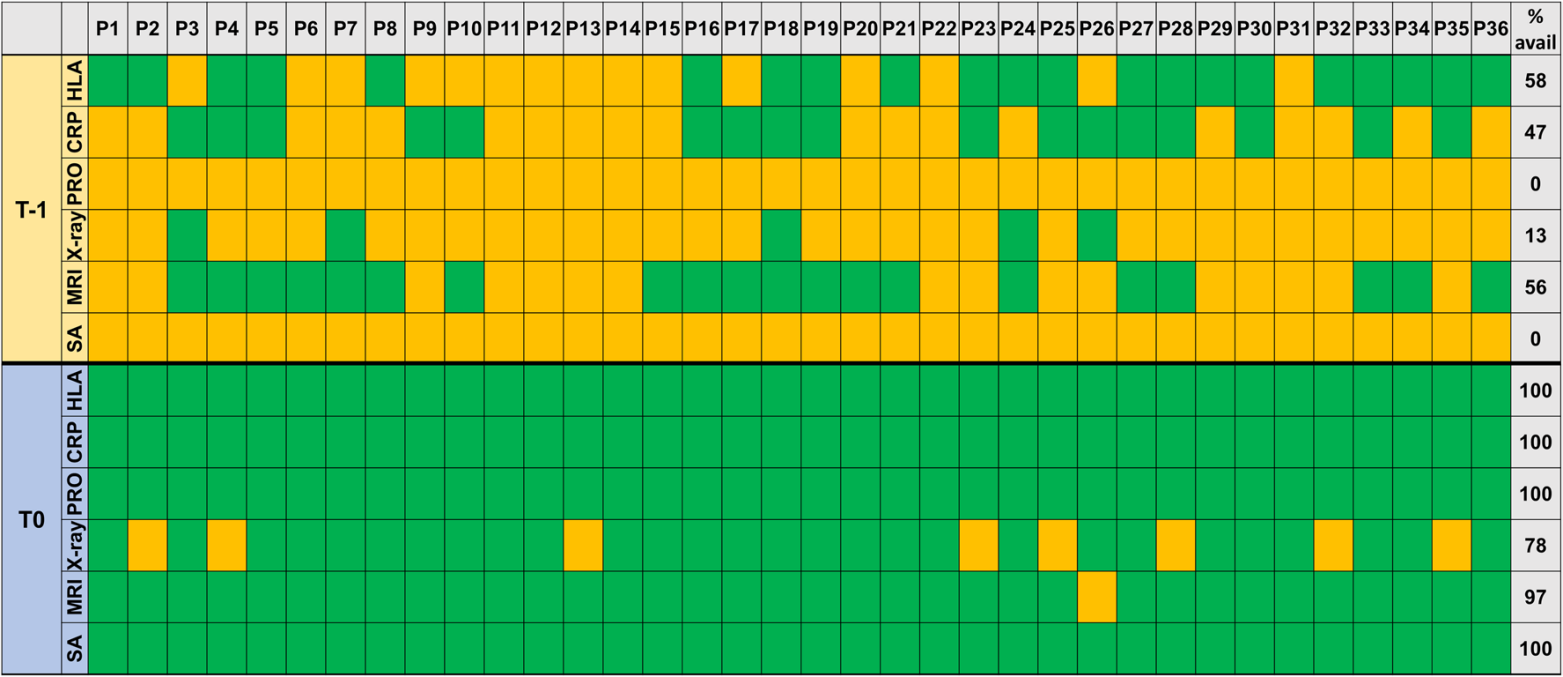
Availability of medical data at T-1 and T0. Total percentage of available results and whether result were available (green) or not (orange) at time points T-1 (student consultation) and T0 (physician consultation). P, patient; SA, structured anamnesis; HLA, HLA-B27; avail, available

At T-1, CRP level had not been assessed in 19/36 (53%) patients, and no sacroiliac joint X-ray and lumbar/sacroiliac MRI scan was available in 31/36 (86%) and 16/36 (44%) patients, respectively (Fig. 1). Due to the student-led clinics, all diagnostic workup had been completed at T0.

Symptom onset preceded T0 by a median of 889 (1507) days (IQR); see Table 2. 14/36 (39%) of patients checked their symptoms on the internet prior to their appointment, 273 (449) days before T0. Time until first presentation could be reduced significantly from an average of 92 days (T0) to 25 days (T-1); p < 0.0001. Student-led clinics also significantly reduced the time interval from the suspected diagnosis to the first on-site presentation appointment.

**Table 2:** Diagnostic delay

Time parameter	Time (days) until (Med (IQR))		N =	Statistics (p value)
	T-1	T0		
Symptom onset	870 (1476)	889 (1507)	36	0.59
First pres. to any physician	456 (727)	494 (681)	27*	0.44
Date of suspected diagnosis	50 (36)	108 (76)	36	< 0.0001
Internet research	227 (469)	273 (449)	14**	0.36
Appointment request	25 (25)	92 (63)	36	< 0.0001
axSpA therapy initiation	32 (67)	− 22 (34)	17***	< 0.0001

The time intervals of various time parameters up to time point T-1 (student-led visit) and T0 (regular initial physician-led visit) are presented and statistically compared (Mann–Whitney U test). Pres., presentation

* not remembered by all patients

** not performed by all patients

***only axSpA patients included in analysis

**Fig. 2:**
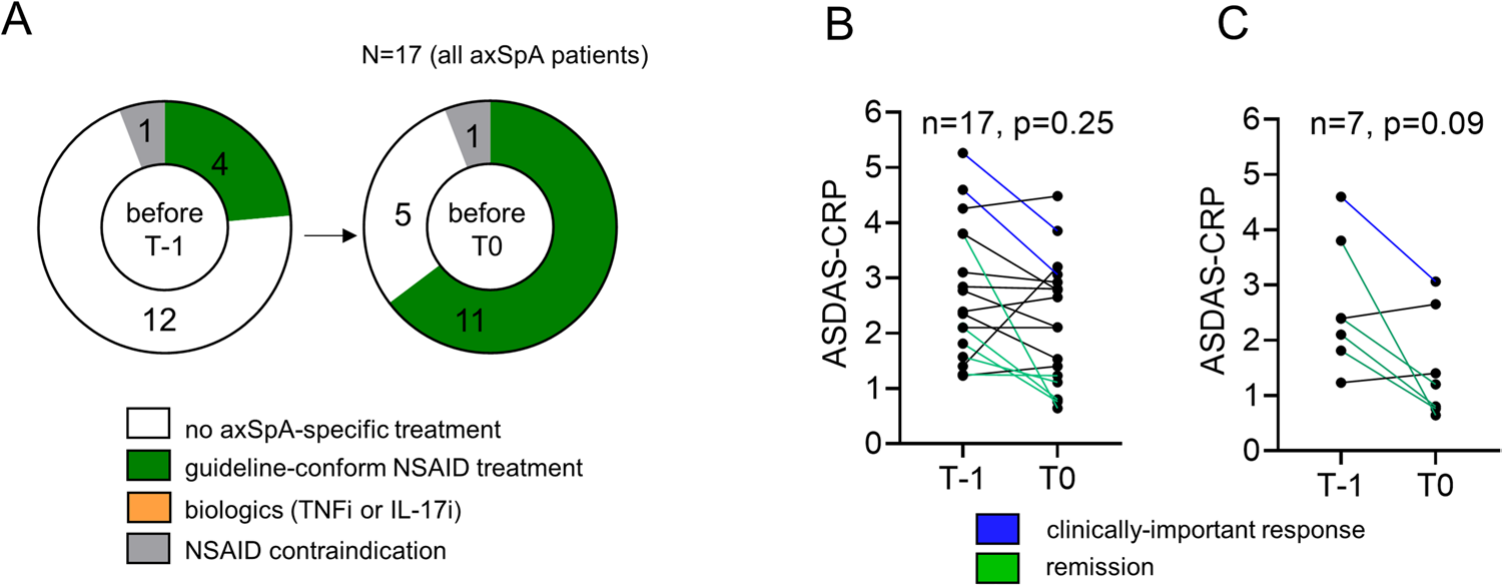
Early initiation of axSpA therapy. **A** The therapies of the 17 axSpA patients at the time before T-1 and before T0 are shown. **B** ASDAS-CRP scores of all axSpA patients and **C** of all newly NSAID-treated axSpA patients at T-1 and T0 are demonstrated. Statistical significance was assessed using the Mann–Whitney U test

Only a minority of patients (4/17, 23.5%, Fig. 2A) had already undergone guideline-conform therapeutic steps of axSpA with two different NSAIDs, each for at least 2 weeks, before first presentation at T-1. NSAID treatment/NSAID rotation, physiotherapy, or app-based physical therapy could be initiated already before T0, as soon as the diagnostics were completed. On average, this allowed guidelines-based NSAID therapy to be initiated significantly earlier with 22 days before T0 in axSpA patients; see Table 2. At T0, 2/17 (11.8%) axSpA patients presented with a clinically important improvement (reduction of ASDAS-CRP ≥ 1.1) and 5/17 (29.4%) patients were in remission (ASDAS-CRP < 1.3); see Fig. 2B. The majority of the 7 NSAID-treated patients at T-1 clinically improved (1/7, 14.3%) or reached remission (4/7, 57.1%) until T0 (Fig. 2C). The student's diagnostic accuracy slightly decreased with physical examination but gradually increased after review of laboratory results and imaging, respectively, to 86.1% (sensitivity 76.5%, specificity 94.7%), online supplemental material S1.

34/36 (94.4%) patients completed at least 80% of the ePROs between T-1 and T0 enabling remote monitoring of disease activity and therapeutic response. Electronic and paper-administered BASDAI correlated well at T0 (r = 0.8 *p* < 0.0001, online supplemental material S2).

Patient acceptance was high for the student-led clinic and ePRO monitoring with a NPS of + 62% (mean ± SD 9.2 ± 0.9) and + 30.5% (mean ± SD 8.0 ± 1.7), respectively, online supplemental material S3.

## Discussion

To our knowledge, this is the first study to examine the added value of student-led clinics and ePROs to accelerate diagnostic workup and treatment in patients with suspected axSpA. Due to the implementation of student-led clinics, patient appointments could be accelerated by more than 2 months, so that all diagnostic workup was complete at the actual rheumatologist visit and some patients already reached the goal of clinical remission. Importantly, patients showed great acceptance and also rheumatologists enjoyed the concept as student-led clinics also drastically reduced their consultation workload. Importantly, the medical student gained valuable clinical experience. The task of a sequential diagnosis (axSpA; yes/no) further supported critical diagnostic thinking. The general increase in diagnostic accuracy with additional information available supports a previous study by Ehrenstein et al. [18], that also examined relative contributions of sequential diagnostic steps. Being limited to medical history data only, they could show that even experienced rheumatologists only reach a diagnostic accuracy of 27%.

Early implementation of ePROs enabled remote, yet standardized assessment of a therapeutic response and importantly timely change of treatment. The concordance of paper-based and electronic BASDAI and high adherence of ePRO monitoring in patients with high disease activity is in line with the previous work [19]. To ensure monitoring adherence also in patients in remission, we believe that there needs to be a clear benefit for patients, such as the optional elimination of a physical appointment. In a large survey, we could previously show that patients generally embrace this concept [20]. Importantly, de Thurah et al. could previously demonstrate in a large randomized-controlled trial (RCT) that ePRO-based monitoring is safe in patients with rheumatoid arthritis [21]. Two large RCTs [22, 23] are currently ongoing and exploring, whether this ePRO-based monitoring is also safe in axSpA patients. In a different study (in review), we could demonstrate that the gold standard for disease activity monitoring, the ASDAS-CRP can be completely carried out at home by patients using a medical app and self-collecting capillary blood for CRP analysis, similar to the previous studies [24, 25].

### Limitations

The small sample size and monocentric nature of the study are clear limitations. The individual student and app used for ePROs likely had a great impact on patient acceptance. The results should, therefore, be confirmed in larger studies at other centers, with different personnel and apps. The general concept could be rolled out to other diseases.

## Conclusion

The need for innovative rheumatology health service strategies is imminent. Implementation of student-led clinics and asynchronous telehealth services, such as ePROs, could contribute to accelerate diagnosis and more efficient use of limited healthcare resources. Analysis of cost-effectiveness and safety will be essential for wider implementation. Additionally, work load reduction and acceptance of physicians should be evaluated in future studies.

## Supplementary Information

Below is the link to the electronic supplementary material.

Supplementary file1 (TIF 209 KB)

Supplementary file2 (TIF 105 KB)

Supplementary file3 (TIF 52 KB)

## Data Availability

Data analyzed during the current study are available from the corresponding author on reasonable request.
