# Supplementary figures and images for: Student-led clinics and ePROs to accelerate diagnosis and treatment of patients with axial spondyloarthritis: results from a prospective pilot study
Source: Rheumatol Int. 2023 Jul 24;43(10):1905–11. doi: 10.1007/s00296-023-05392-5 (PMC10435605; doi:10.1007/s00296-023-05392-5)

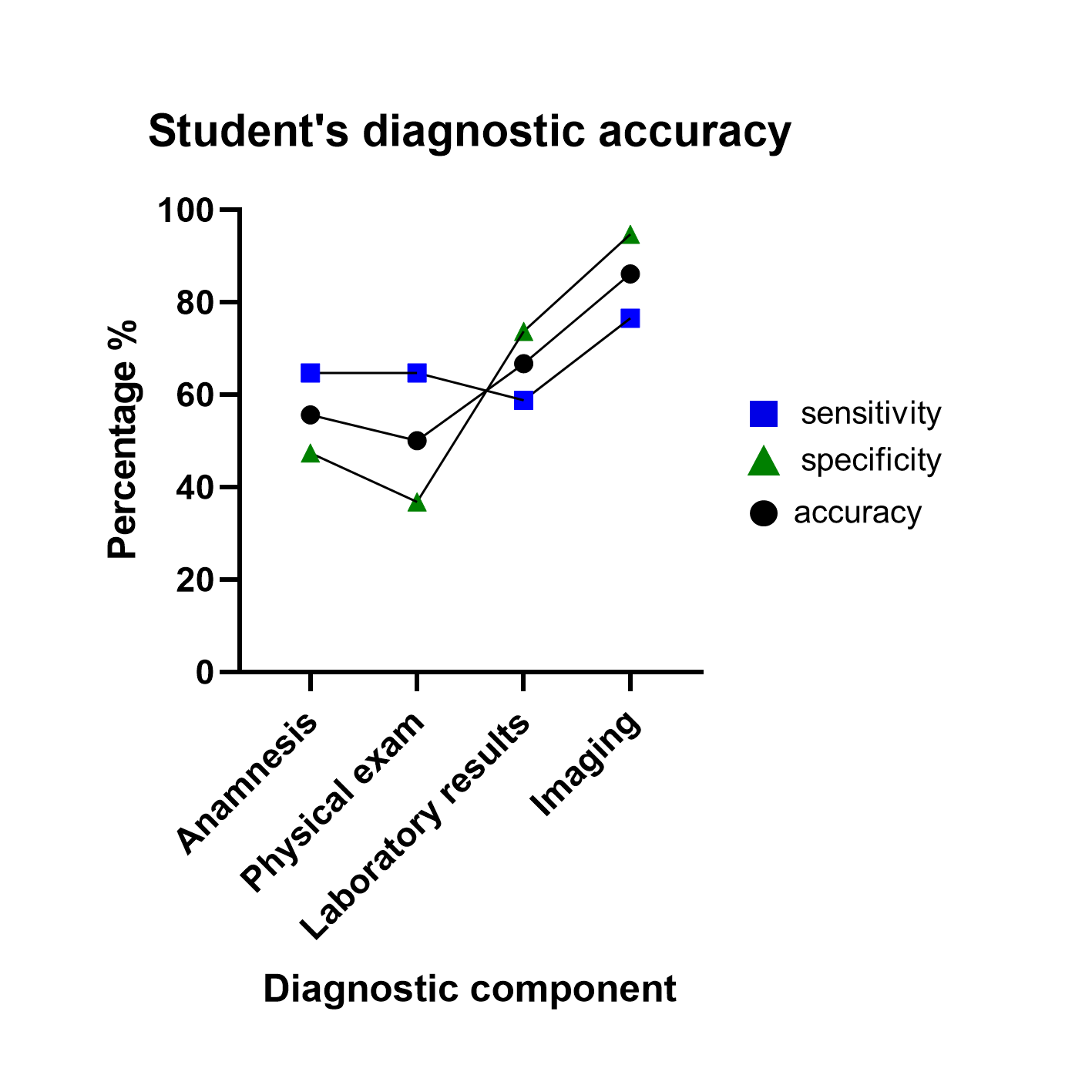

Supplement: Supplementary file 1 — Supplementary file1 (TIF 209 KB) [file 296_2023_5392_MOESM1_ESM.tif]

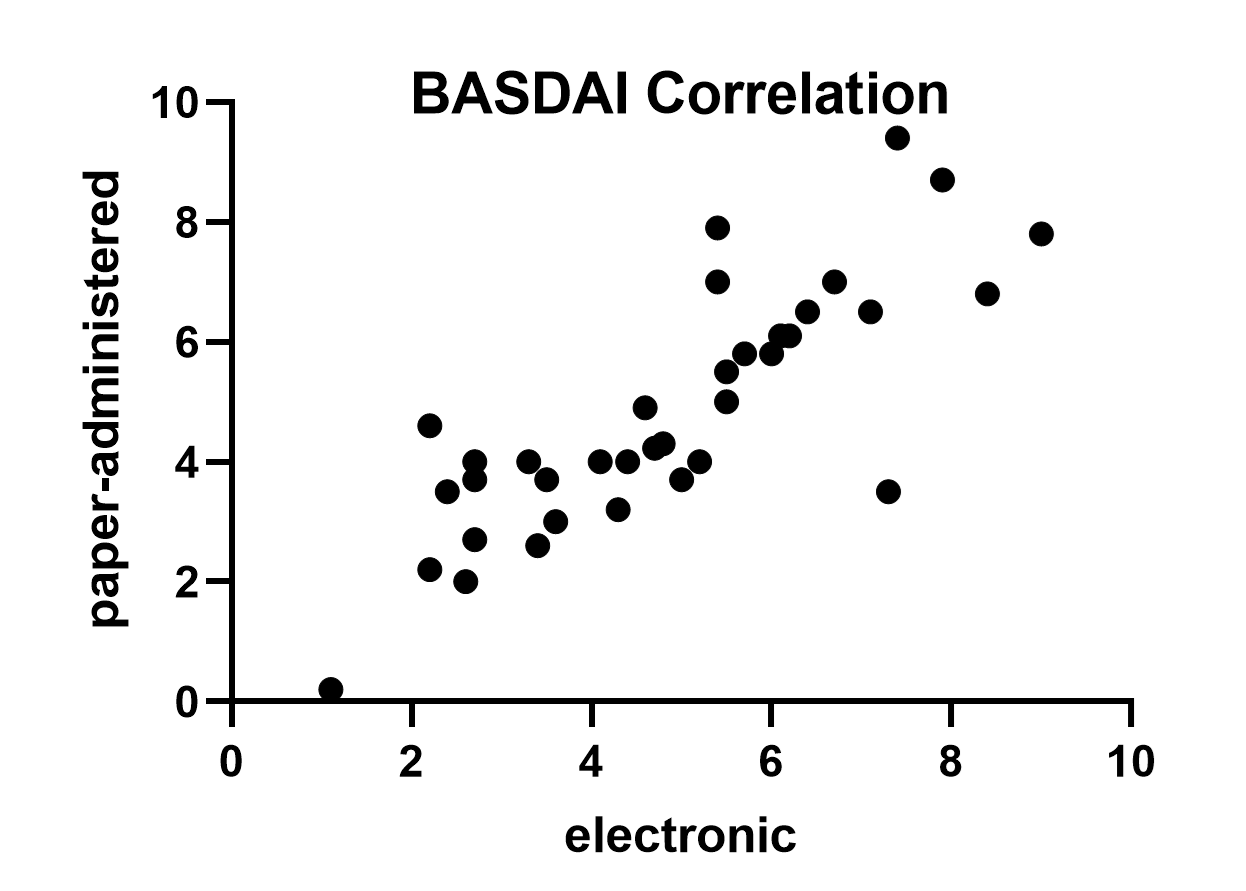

Supplement: Supplementary file 2 — Supplementary file2 (TIF 105 KB) [file 296_2023_5392_MOESM2_ESM.tif]

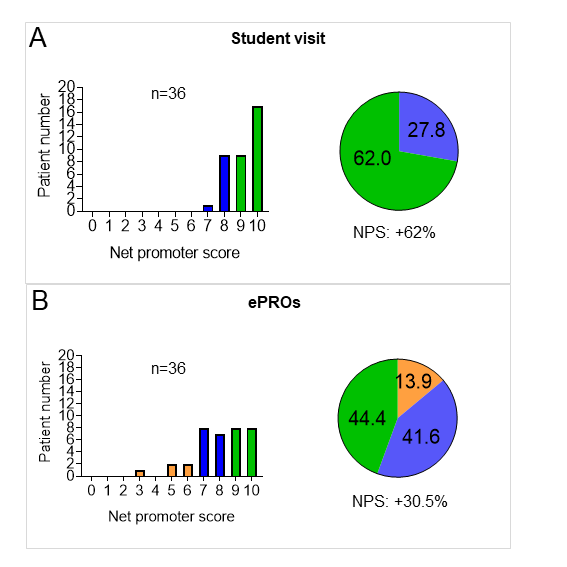

Supplement: Supplementary file 3 — Supplementary file3 (TIF 52 KB) [file 296_2023_5392_MOESM3_ESM.tif]
